# Yijing Decoction improves oocyte quality and inhibits lactate accumulation in mice with diminished ovarian reserve

**DOI:** 10.3389/fmolb.2026.1946851

**Published:** 2026-10-15

**Authors:** Jiaqi Yang, Mengyuan Chu, Han Zhang, Wenman Meng, Chi Zhang, Yunfei Han, Genping Zeng, Qianyi Zhou, XiaoYu Zhang, Fengrong Zhang, Ying Yan

**Affiliations:** 1 First Teaching Hospital of Tianjin University of Traditional Chinese Medicine, Tianjin, China; 2 National Clinical Research Center for Chinese Medicine, Tianjin, China

**Keywords:** diminished ovarian reserve, lactate, oocyte, SIRT3/HIF-1α/PDK1, YJD

## Abstract

**Background:**

Diminished ovarian reserve (DOR) is characterized by a decline in both oocyte quantity and quality, leading to compromised fertility. Yijing Decoction (YJD) has demonstrated promising therapeutic potential in DOR management. Nevertheless, the mechanisms driving this phenomenon are still largely unclear. This study is designed to comprehensively assess the effects of YJD on ovarian reserve function and oocyte quality in DOR mice, while investigating its potential underlying mechanisms of action.

**Methods:**

A DOR mouse model was established by administering cyclophosphamide via a single intraperitoneal injection. The therapeutic efficacy of YJD was systematically assessed through multiple functional and histological parameters, including ovarian index, estrous cycle regularity, serum sex hormone levels (FSH, LH, E_2_, AMH, and INH-B), and follicle counts, as well as the number, quality, and spindle morphology of metaphase I (MI) oocytes. To elucidate the underlying molecular mechanisms, ATP levels, pyruvate uptake, and lactate production were further quantified using commercially available ATP, pyruvate, and lactate assay kits. Additionally, immunofluorescence microscopy was employed to assess the protein expression levels of glycolysis-related proteins, including GLUT1, GLUT4, and LDHA, as well as components of the SIRT3/HIF-1α/PDK1 signaling axis in MI oocytes.

**Results:**

YJD treatment improved ovarian reserve function and oocyte quality in DOR mice, as evidenced by increased ovulated oocyte numbers, improved spindle integrity, and restored ATP levels. In addition, YJD treatment was associated with reduced expression of glycolysis-related proteins, including GLUT1, GLUT4, and LDHA, as well as decreased pyruvate uptake and lactate production in MI oocytes. YJD treatment also partially restored the expression pattern of the SIRT3/HIF-1α/PDK1 signaling pathway in DOR oocytes.

**Conclusion:**

YJD improves oocyte quality in DOR mice, and its protective effects may be associated with the regulation of lactate metabolism and the SIRT3/HIF-1α/PDK1 signaling pathway.

## 1 Introduction

Diminished ovarian reserve (DOR), defined by a reduction in both the quantity and quality of oocytes, is a leading cause of ovulatory dysfunction-associated infertility in women (Sharara et al., 1998). The prevalence of DOR has increased markedly from 19% in 2004 to 26% in 2014, with a growing trend toward earlier onset (Hosseinzadeh et al., 2023; Jiao and Bukulmez, 2021; Liu et al., 2025). Declining oocyte quality constitutes the central pathological mechanism underlying DOR, directly contributing to suboptimal embryo development and elevated rates of early pregnancy loss, which ultimately reduce clinical pregnancy and live birth rates (An et al., 2024; Lin et al., 2024; Cedars, 2022; Jaswa et al., 2021). Given the profound impact of DOR on female fertility, this condition can no longer be overlooked. Therefore, elucidating the cellular mechanisms that contribute to the deterioration of oocyte quality is essential for identifying effective therapeutic targets for DOR.

Increasing evidence suggests that dysregulated lactate metabolism may be closely associated with impaired oocyte quality. Lactate is generated from pyruvate by lactate dehydrogenase A (LDHA) and serves as an important metabolite involved in glycolysis, cellular redox balance, and energy metabolism (Wu et al., 2021). Under physiological conditions, lactate production contributes to the maintenance of metabolic homeostasis; however, excessive diversion of pyruvate toward lactate may reflect disturbed glucose metabolism and reduced mitochondrial utilization of pyruvate (Li et al., 2026). This metabolic imbalance is particularly relevant to oocyte maturation, which requires sufficient ATP to support meiotic progression, spindle assembly, and chromosome segregation. Insufficient ATP availability has been associated with meiotic abnormalities, spindle defects, and chromosomal aneuploidy, ultimately compromising oocyte quality (Wang et al., 2025; Li et al., 2022). Notably, metabolomic analysis of human oocytes has shown that aging is accompanied by increased pyruvate and lactate levels and impaired mitochondrial metabolism, particularly at the metaphase I (MI) stage (Smits et al., 2023). In addition, recent studies in patients with DOR have revealed disturbances in glucose metabolism and lactate-related metabolic pathways in ovarian granulosa cells, further supporting an association between altered lactate metabolism and impaired ovarian function (Zhu et al., 2025). Experimental evidence also indicates that excessive LDHA-mediated conversion of pyruvate to lactate is associated with reduced ATP availability and impaired developmental competence, whereas correction of this metabolic imbalance improves reproductive outcomes (Yang et al., 2025). Collectively, these findings suggest that abnormal lactate accumulation and dysregulated pyruvate-to-lactate metabolism may constitute an important metabolic mechanism contributing to reduced oocyte quality in DOR.

Pyruvate dehydrogenase kinase 1 (PDK1) is a critical regulatory enzyme in glucose metabolism that phosphorylates and inhibits the pyruvate dehydrogenase complex, thereby suppressing the conversion of pyruvate to acetyl-CoA and diverting glycolytic flux toward lactate production. This metabolic reprogramming reduces mitochondrial oxidative phosphorylation and compromises cellular ATP generation efficiency (Lin et al., 2024). Hypoxia-inducible factors (HIFs) are master transcriptional regulators of cellular metabolic adaptation. Specifically, hypoxia inducible factor-1alpha (HIF-1α) functions as a potent transcriptional activator of multiple glycolytic genes, directly upregulating the expression of PDK1, LDHA, and other glycolysis-associated proteins (Morris et al., 2022). Recent studies have demonstrated that in cyclophosphamide (CTX)-induced DOR mice, HIF-1α protein levels are significantly elevated in both ovarian tissue and oocytes (Zhu et al., 2022; Wang et al., 2026). Sirtuin 3 (SIRT3), a major NAD^+^-dependent mitochondrial deacetylase, negatively regulates glycolysis by promoting HIF-1α degradation (Zhang et al., 2026). Genetic ablation of SIRT3 results in HIF-1α stabilization and a consequent metabolic shift toward HIF-1α–dependent glycolysis (Wang et al., 2019; Chang et al., 2021). Clinically, in vitro–immature oocytes exhibit significantly lower SIRT3 mRNA expression compared with their in vitro–matured (IVM) counterparts; moreover, experimental overexpression of SIRT3 has been shown to improve oocyte developmental competence (Zhao et al., 2016). Taken together, these findings suggest that dysregulation of the SIRT3/HIF-1α/PDK1 axis may promote excessive pyruvate-to-lactate conversion and lactate accumulation, thereby contributing to impaired oocyte quality in DOR.

Yijing Decoction (YJD) is a classical traditional Chinese medicine (TCM) formula originating from the Qing Dynasty and first documented by Fu Shan in Fu Qingzhu’s Gynecology. Its composition includes processed Rehmanniae radix praeparata (ShuDiHuang), Atractylodes macrocephala koidz. (BaiZhu), Rhizoma dioscoreae (ShanYao), Angelicae sinensis radix (DangGui), Paeoniae radix alba (BaiShao), Ziziphi spinosae semen (SuanZaoRen), Moutan cortex (MuDanPi), Glehniae radix (BeiShaShen), Radix bupleuri (ChaiHu), Eucommiae Cortex (DuZhong), and Panax ginseng C.A.Mey. (RenShen). Clinically, YJD is employed to tonify the kidney, regulate liver function, nourish blood, and normalize menstruation. Clinical studies have confirmed that YJD increases the number of antral follicles, retrieved oocytes, mature oocytes, and high-quality embryos, thereby improving *In vitro* fertilization (IVF) outcomes in older patients. Network pharmacology analyses suggest that YJD exerts its protective effects on ovarian function through multi-target modulation of key biological pathways, including oxidative stress and angiogenesis (Gao et al., 2025; Xu et al., 2025). Modern pharmacological investigations further reveal that Angelicae sinensis radix, a core constituent of YJD, reduces lactate accumulation (Zhong et al., 2016), whereas Rehmanniae radix praeparata and Eucommiae cortex achieve similar effects via inhibition of glycolytic activity (Zhang et al., 2026; Park et al., 2006). These observations raise the possibility that regulation of abnormal lactate metabolism may represent an important mechanism underlying the beneficial effects of YJD on ovarian function. Nevertheless, the precise molecular mechanisms underlying YJD’s therapeutic efficacy against DOR warrant further systematic investigation.

In this study, a DOR mouse model was established via CTX administration, and the therapeutic effects of YJD on restoring ovarian reserve function in DOR mice were systematically evaluated. To further validate YJD’s impact on oocyte quality in DOR mice, oocyte retrieval at the MI stage was performed, followed by immunofluorescent staining of the meiotic spindle. Subsequently, measurements of ATP levels, pyruvate uptake, lactate production, and the expression of glycolysis-related proteins and the SIRT3/HIF-1α/PDK1 pathway was quantified to mechanistically assess YJD’s regulatory role in glycolytic flux and lactate accumulation in MI oocytes.

## 2 Materials and methods

### 2.1 Preparation of YJD extract

The preparation of YJD followed the traditional decoction method commonly used in clinical practice in TCM. All herbs (listed in Table 1) were procured from the Chinese Herbal Medicine Dispensary of the First Affiliated Hospital of Tianjin University of Traditional Chinese Medicine. Briefly, the herbs were soaked in distilled water for 30 min, then transferred to a decoction pot with additional distilled water, brought to a boil, and subsequently simmered at low heat for 60 min to yield the first decoction. The residue was re-extracted by adding fresh distilled water, bringing to a boil, and simmering for another 60 min to obtain the second decoction. The two decoctions were pooled, concentrated under reduced pressure using rotary evaporation to a final concentration of 1.15 g/mL (expressed as crude herb weight per mL), and stored at 4 °C until further use.

**TABLE 1:** Composition of Yijing decoction.

Components	Chinese name	Used part	Weight (g)
Rehmanniae radix praeparata	ShuDiHuang	Root	30
Atractylodes macrocephala koidz	BaiZhu	Root and rhizome	30
Rhizoma dioscoreae	ShanYao	Root and rhizome	15
Angelicae sinensis radix	DangGui	Root	15
Paeoniae radix alba	BaiShao	Root	9
Ziziphi spinosae semen	SuanZaoRen	Seed	9
Moutan cortex	MuDanPi	Root	6
Glehniae radix	BeiShaShen	Root	9
Radix bupleuri	ChaiHu	Root	3
Eucommiae cortex	DuZhong	Bark	3
Panax ginseng C. A. Mey	RenShen	Root and rhizome	6

The chemical profile and blood-penetrating constituents of YJD have been comprehensively characterized in our previous investigations, and the relevant analytical results are summarized in Supplementary Tables S1 and S2. The putative herbal source(s) of each identified constituent are also provided in Supplementary Tables S1 and S2 based on information obtained from HERB and TCMSP database. Because some constituents may be present in more than one herb, these source assignments should be regarded as putative rather than definitive. The chemical characterization was intended to define the compositional basis of YJD, whereas direct associations between individual constituents and the biological effects observed in the present study remain to be established.

### 2.2 Animals and administration

A total of 80 female ICR mice (6–8 weeks old, weighing 20–30 g) were obtained from Yishengyuan Gene Technology (Tianjin) Co., Ltd. (Tianjin, China) (Certificate No.: SYXK (Tianjin) 2021–0003). The experimental protocol was approved by the Institutional Animal Care and Use Committee of Yishengyuan Gene Technology (Tianjin) Co., Ltd. (Approval No. YSY-DWLL-2024377). All experimental animals were maintained in standard controlled conditions.

After 7 days of acclimatization and consecutive vaginal smear examinations, 72 female mice exhibiting regular estrous cycles were selected for the formal experiment. These animals were randomly assigned to six experimental groups (n = 12 per group): the control group, the model group, the YJD low-dose group (YJD-L), the YJD medium-dose group (YJD-M), the YJD high-dose group (YJD-H), and the estradiol valerate group (EV). To establish the diminished ovarian reserve (DOR) model, all mice except those in the control group received a single intraperitoneal injection of cyclophosphamide (31626F, Baxter Oncology GmbH, Germany) at a dose of 75 mg/kg (Li et al., 2024). Successful model induction was confirmed by the onset of persistent estrous cycle irregularities (Supplementary Figure S1). Subsequently, pharmacological interventions commenced. Doses for the YJD and EV groups were determined by converting human clinical daily doses into mouse-equivalent doses using body surface area normalization (Lin et al., 2026). Specifically, mice in the YJD-L, YJD-M, and YJD-H groups received oral gavage of YJD suspension at 0.9 g/kg, 1.8 g/kg, and 3.6 g/kg—corresponding to 0.5×, 1×, and 2× the respective human equivalent doses, while mice in the EV group received oral gavage of estradiol valerate tablets (Bayer, 756A) at 0.09 mg/kg. Concurrently, mice in the control and model groups received an equivalent volume of vehicle (sterile water) via oral gavage. After 8 weeks of intervention, mice were anesthetized by intraperitoneal injection of 2% pentobarbital sodium (40 mg/kg), and blood samples were collected. Subsequently, the mice were euthanized by cervical dislocation, and ovarian tissues and MI oocytes were harvested.

### 2.3 Body weight, uterine and ovarian index, and estrous cycle evaluation

During the experiment, body weight was recorded weekly for each animal. At the end of the intervention, body weight, uterine wet weight, and ovarian wet weight were measured for each mouse. Subsequently, the uterine and ovarian indices were determined as percentages using the equation: Uterine or ovarian index (%) = [wet weight of uterus or ovaries (g)/body weight (g)] × 100%.

### 2.4 Estrus cycle detection

At 24 h after model induction and 14 days prior to the end of treatment, vaginal smears were collected daily from mice between 9:00 a.m. and 10:00 a.m. for 14 consecutive days using sterile, pointed medical swabs. Estrous cycle staging was determined by light microscopic examination (OLYMPUS, Tokyo, Japan) of cellular morphology in the smears: inter-estrus was characterized predominantly by leukocytes; proestrus by nucleated epithelial cells; estrus by anucleated, keratinized epithelial cells; and diestrus by a mixture of nucleated epithelial cells, anucleated keratinized epithelial cells, and leukocytes.

### 2.5 Histological analysis and follicle counting

Ovarian specimens were immersed in a 4% paraformaldehyde solution for 24 h, then subjected to routine dehydration procedures and embedded in paraffin wax. Five-micrometer-thick histological sections were cut from the embedded tissue. After dewaxing and rehydration, sections underwent hematoxylin and eosin (H&E) staining. Digital images of the stained sections were acquired and analyzed using a digital slide scanning system (3DHISTECH, Hungary). Primordial, primary, secondary, antral, and atretic follicles recognized based on established morphological criteria (Hu et al., 2025).

### 2.6 Measurement of hormone levels

Serum was separated from mouse blood by centrifugation at 3,500 rpm for a duration of 15 min. Serum concentrations of follicle-stimulating hormone (FSH, F2555-A, Shfksc, Guangzhou, China), luteinizing hormone (LH, F2582-A, Shfksc, Guangzhou, China), estradiol (E_2_, F2566-A, Shfksc, Guangzhou, China), anti-Müllerian hormone (AMH, F9402-A, Shfksc, Guangzhou, China), and inhibin B (INH-B, F2507-B, Shfksc, Guangzhou, China) were quantified using commercially available enzyme-linked immunosorbent assay (ELISA) kits (Shfksc, Guangzhou, China). Absorbance was measured at 450 nm using an ELISA microplate reader (Beijing Perlong New Technology Co., Ltd., Beijing, China), and hormone concentrations were calculated according to the manufacturer’s instructions.

### 2.7 Extraction of MI oocytes

To retrieve MI oocytes, mice were administered 5 IU of pregnant mare serum gonadotropin (PMSG, 240,602, Tianjin Zhengjiang Modern Biotechnology, Tianjin, China) via intraperitoneal injection. Forty-eight hours later, mice were administered 5 IU of human chorionic gonadotropin (hCG, A2405311, Ningbo Second Hormone Co., Ltd., Tianjin, China) by the same route. After 6–7 h, the mice were euthanized. Bilateral oviducts were excised and immediately transferred into pre-warmed M2 medium. Under a stereomicroscope, the dilated ampullary region of each oviduct was punctured using a fine glass needle. Cumulus–oocyte complexes (COCs) were gently flushed out and collected in M2 medium. COCs were then incubated in 2 mg/mL hyaluronidase (BS171-100 mg, Biosharp, Beijing, China) for 30 min at 37 °C to remove cumulus cells. After three consecutive washes with M2 medium, oocytes were counted and imaged under an inverted microscope (Thermo Fisher Scientific, China).

### 2.8 Pyruvate uptake and lactate production

Collect 150–200 oocytes per experimental group. Immediately transfer the oocytes into 50-μL droplets of pre-warmed M16 medium, overlay each droplet with mineral oil, and incubate at 37 °C under 5% CO_2_ for 4 h.

Pyruvate uptake is determined as the difference in pyruvate concentration between the blank (i.e., fresh, unconditioned) medium and the waste (i.e., oocyte-conditioned) medium. Using the pyruvate assay kit (S0299S, Beyotime Biotechnology, Shanghai, China) and following the manufacturer’s instructions, prepare a standard curve using pyruvate standards at final concentrations of 0, 10, 20, 50, 100, and 200 μM. In a 96-well black microplate, dispense 20 μL of Pyruvate Assay Buffer, 20 μL of each standard solution, 20 μL of blank medium, and 20 μL of waste medium into wells designated as “Blank,” “Standard,” and “Test,” respectively. Add 80 μL of Amplex Red Reaction Buffer to each well, and incubate the plate at 37 °C in the dark for 10 min. Measure fluorescence intensity using an excitation wavelength of 560 nm and an emission wavelength of 590 nm.

Lactate levels were quantified with a commercially available lactate detection kit (S0227S, Beyotime Biotechnology, Shanghai, China), according to the protocol provided by the manufacturer. A series of calibration solutions were prepared with concentrations spanning 0, 1, 2, 5, 10, and 20 μM. Into a 96-well black microplate, 50 μL of Lactate Assay Buffer, 50 μL of each standard solution, 50 μL of blank medium, and 50 μL of waste medium were dispensed into wells labeled “Blank,” “Standard,” and “Sample,” respectively. Next, 50 μL of the Amplex Red reaction buffer was dispensed into each well, followed by a 30-min incubation at 37 °C under light-protected conditions. Fluorescence signals were then quantified with an excitation wavelength of 560 nm and an emission wavelength of 590 nm. Lactate production was calculated as the difference in fluorescence signal between the waste medium and the blank medium.

### 2.9 ATP assay

Following the manufacturer’s instructions, intracellular ATP levels in oocytes were quantified using an ATP assay kit (S0027, Beyotime Biotechnology, Shanghai, China). For each experimental group, 50–100 MI oocytes were lysed in 100 µL of lysis buffer. A standard curve was generated using ATP standards prepared at concentrations of 0.01, 0.03, 0.1, 0.3, 1, 3, and 10 µM. Both samples and standards were loaded into a 96-well black microplate. Luminescence intensity was measured using an Infinite M200 Pro microplate reader (Tecan Group Ltd., Männedorf, Switzerland). ATP concentrations in the samples were determined by interpolation from the standard curve.

### 2.10 Immunofluorescence staining

Oocytes were initially fixed using a 4% paraformaldehyde solution for 30 min at ambient temperature. Following fixation, specimens underwent three rinses in phosphate-buffered saline (PBS). Next, membrane permeabilization was achieved by incubating the oocytes in PBS containing 0.5% Triton X-100 for 60 min under room-temperature conditions. After another three PBS washes, nonspecific antibody binding sites were saturated by incubating samples in a blocking buffer composed of 1% bovine serum albumin (BSA) for 60 min at room temperature. Primary antibodies—diluted in the same blocking solution—were then applied overnight at 4 °C. The antibodies used included: anti-β-tubulin (1:200, AF7010, Affinity Biosciences, Liyang, China), anti-PDK1 (1:200, ET1704-66, HUABIO, Hangzhou, China), anti-LDHA (1:500, HA723547, HUABIO, Hangzhou, China), anti-GLUT1 (1:500, ET1601-10, HUABIO, Hangzhou, China), anti-GLUT4 (1:200, R1402-3, HUABIO, Hangzhou, China), anti-SIRT3 (1:100, HA722251, HUABIO, Hangzhou, China), and anti-HIF-1α (1:200, ER1802-41, HUABIO, Hangzhou, China).

The next day, oocytes were rinsed three times in PBS supplemented with 0.01% Triton X-100. Subsequently, they were exposed to a FITC-labeled goat anti-rabbit IgG secondary antibody (1:50, ZF-0311, ZSGB-BIO) for 60 min at 37 °C. After another three PBS rinses, nuclear DNA was visualized by staining with a commercially available DAPI solution (C0065, Beijing Solabio Technology Co., Ltd., Beijing, China) for 30 min under ambient conditions. Following a final series of three PBS washes, the oocytes were placed onto a glass-bottom confocal dish in a 50 μL mounting drop and examined using an inverted laser scanning confocal microscope (Zeiss LSM 900, Germany).

### 2.11 Statistical analysis

Data are expressed as the mean ± SD. To compare groups, one-way ANOVA was applied; subsequently, pairwise comparisons were conducted using the LSD *post hoc* test when data met both normality and homoscedasticity assumptions, or the Dunnett’s T3 test when the assumption of equal variances was violated. Statistical significance was defined as *P* < 0.05.

## 3 Result

### 3.1 YJD improves the ovarian index and estrous cycle in DOR mice

The experimental timeline of YJD’s regulatory effects on ovarian function is illustrated in Figure 1A. No statistically significant variation in initial body weight was detected across experimental groups before treatment initiation. By days 36 and 71, the model group had significantly lower body weight than the control group. In comparison with the model group, administration of high-dose YJD or estradiol valerate elicited a robust and statistically significant elevation in body weight (Figure 1B). Relative to the control group, the model group exhibited a profound and highly significant diminution in ovarian index. Conversely, treatment with medium-dose YJD, high-dose YJD, or estradiol valerate markedly increased ovarian indices (Figures 1C,D). No appreciable intergroup variation in uterine index was observed among any of the experimental cohorts (Figure 1E). Vaginal cytology analysis revealed disrupted estrous cycles in the model group characterized primarily by prolonged inter-estrous intervals, which were ameliorated following treatment with high-dose YJD or estradiol valerate (Figures 1F–I).

**FIGURE 1:**
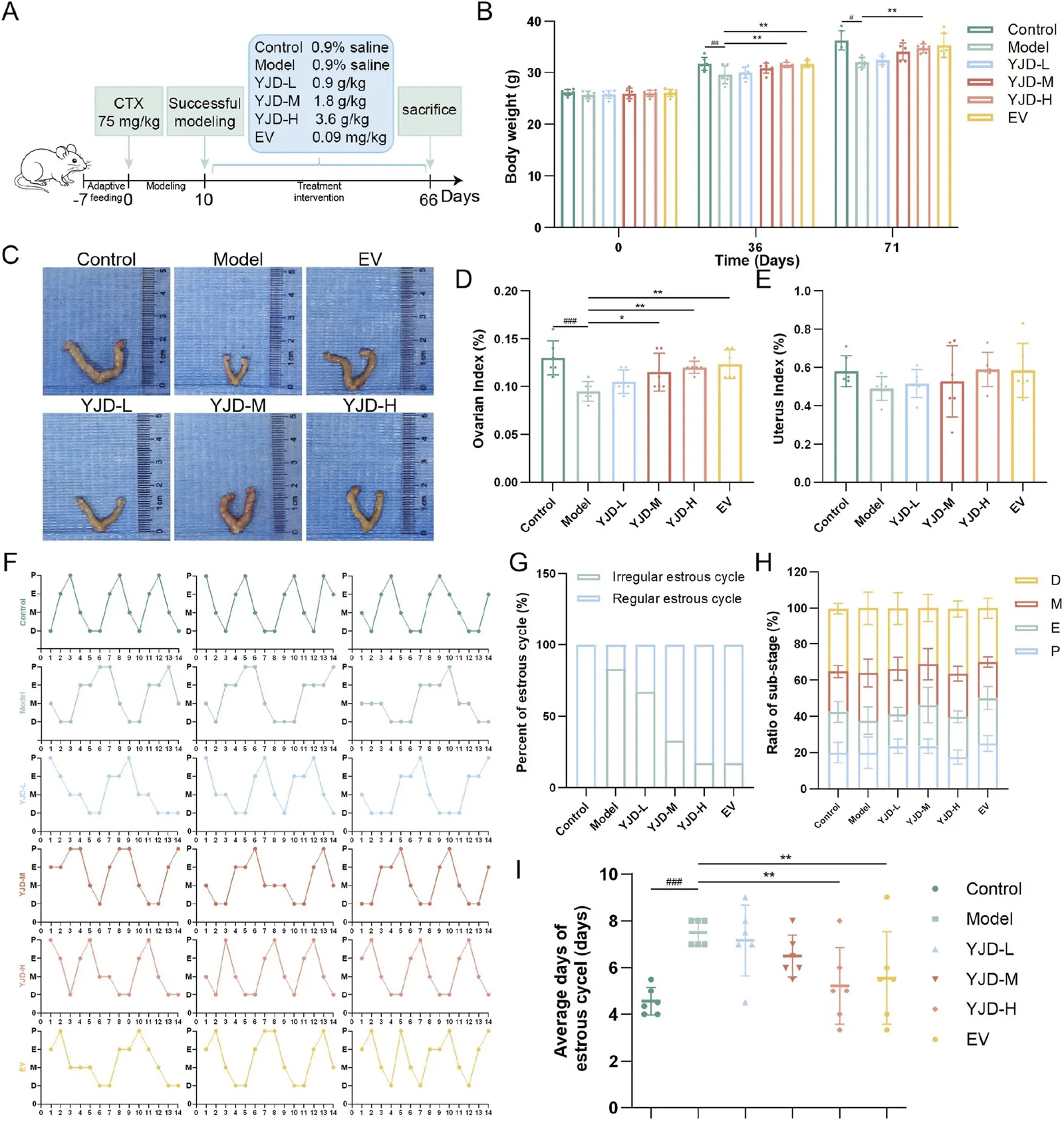
Effects of YJD on ovarian function in DOR mice. **(A)** Schematic diagram of the experimental procedure. **(B)** Body weight at D0, D5, and D10. **(C)** Representative anatomical images of the uterus and ovaries. **(D)** Ovarian index. **(E)** Uterine index. **(F)** Representative estrous cycles of mice. P: Proestrus; E: Estrus; M: Metestrus; D: Diestrus. **(G)** The percent of regular and irregular estrous cycle. **(H)** Proportion of time spent in estrus. **(I)** The average days of estrous cycle. B, D, E, G, H, I: *n* = 6. vs. Control: ^#^ *P* < 0.05, ^##^ *P* < 0.01, ^###^ *P* < 0.001; vs. Model: ^*^ *P* < 0.05, ^**^ *P* < 0.01, ^***^ *P* < 0.001.

### 3.2 YJD improves ovarian reserve function in DOR mice

Serum hormone concentrations were quantified to comprehensively evaluate ovarian endocrine functionality across distinct murine cohorts. Strikingly, the model group exhibited marked endocrine dysregulation. Serum FSH and LH levels surged significantly relative to the control group, whereas E_2_, AMH, and INH-B levels plummeted markedly. In contrast, therapeutic intervention yielded compelling restorative effects. Specifically, serum FSH concentrations were robustly suppressed in the YJD-M, YJD-H, and EV groups versus the model group; E_2_ levels rose appreciably in the YJD-H group; AMH levels were elevated in the YJD-M, YJD-H, and EV groups; and INH-B levels increased significantly in the YJD-M, YJD-H, and EV groups (Figures 2A–E). Ovarian reserve function was further assessed by quantifying follicles at distinct developmental stages in ovarian tissue (Mantawy et al., 2019). Mice in the control group displayed normal ovarian morphology and follicle counts across all developmental stages. In contrast, the model groups exhibited significant reductions in the numbers of primordial follicles, primary follicles, and mature follicles (*P* < 0.01), whereas the numbers of atretic follicles and corpora lutea were significantly elevated. Following high-dose YJD treatment, the numbers of primordial and mature follicles were significantly restored, while the numbers of atretic follicles and corpora lutea were markedly reduced (Figures 2F,G). Collectively, these results demonstrate that YJD ameliorates ovarian reserve dysfunction in DOR mice.

**FIGURE 2:**
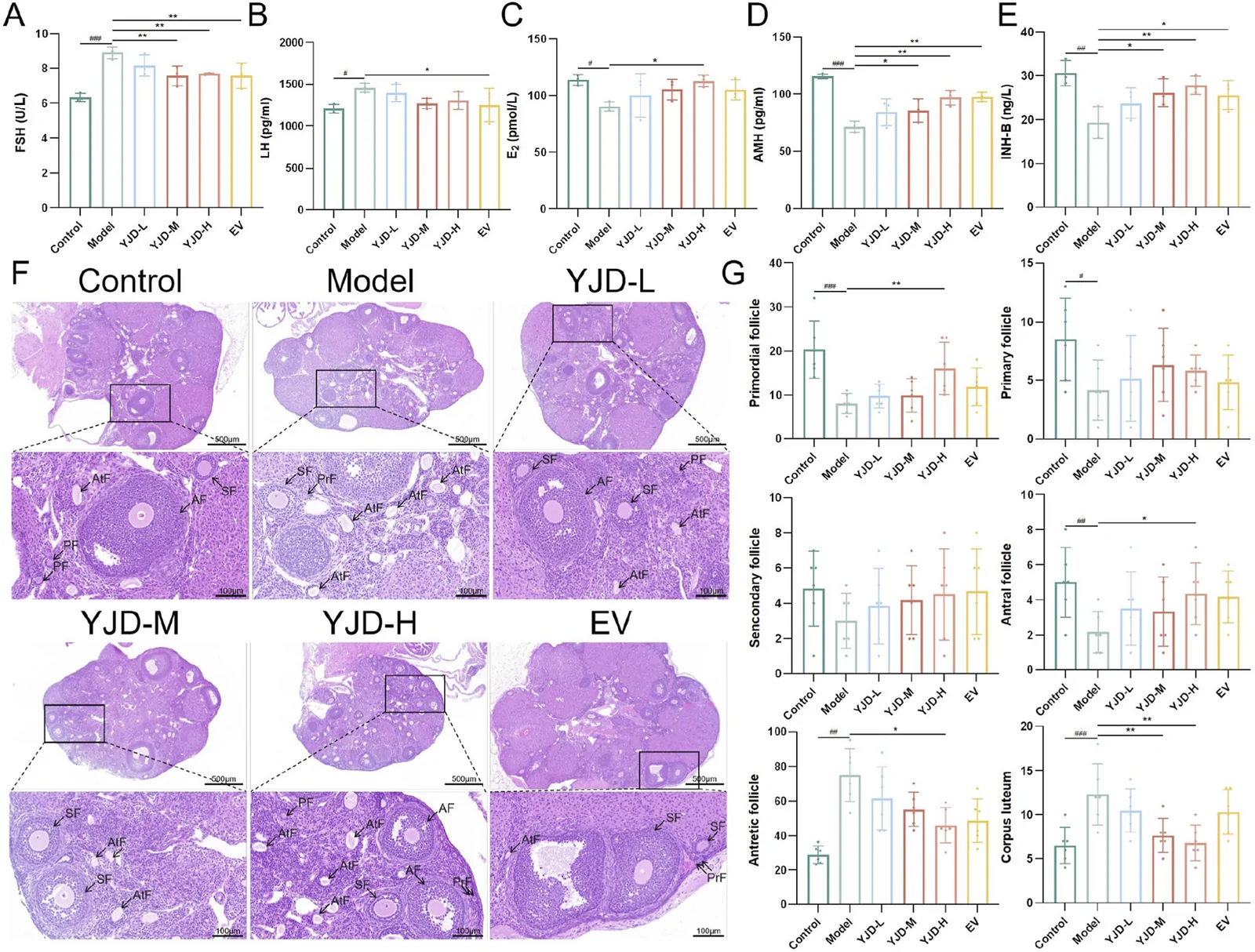
Regulation of ovarian reserve function in DOR mice by YJD. **(A–E)** Serum levels of FSH, LH, E_2_, AMH, and INH-B. **(F)** Representative HE-stained ovarian sections from each group (scale bar: 500 μm, 100 μm). PrF, primordial follicle; PF, primary follicle; SF, secondary follicle; AF, antral follicle; AtF, atresia follicle; CL, corpus luteum. **(G)** Follicle Count by Stage. A–E: *n* = 3; G: *n* = 6. vs. Control: ^#^ *P* < 0.05, ^##^ *P* < 0.01, ^###^ *P* < 0.001; vs. Model: ^*^ *P* < 0.05, ^**^ *P* < 0.01, ^***^ *P* < 0.001.

### 3.3 YJD improves oocyte quality and increases ATP levels

In contrast to the control group, the model group exhibited a significantly reduced oocyte count, aberrant oocyte morphology, an increased proportion of fragmented oocytes, and diminished oocyte quality. Treatment with high-dose YJD or estradiol valerate significantly improved oocyte quality; moreover, high-dose YJD also significantly increased the number of ovulated oocytes (Figures 3A–C). Proper assembly and migration of bipolar spindles represent critical events during MI oocyte maturation, directly influencing oocyte quality and subsequent developmental competence (Yue et al., 2024). This process is highly energy-dependent and requires substantial ATP production and consumption. Consistent with this, oocytes from the model group displayed severe spindle abnormalities and significantly lower intracellular ATP levels relative to the control group (*P* < 0.001). Following treatment with medium-dose YJD, high-dose YJD, or estradiol valerate, the majority of oocytes regained typical barrel-shaped bipolar spindle, and intracellular ATP levels were significantly restored (Figures 3D,E). Collectively, these findings demonstrate that YJD enhances oocyte quality and spindle integrity in DOR mice and effectively upregulates ATP levels.

**FIGURE 3:**
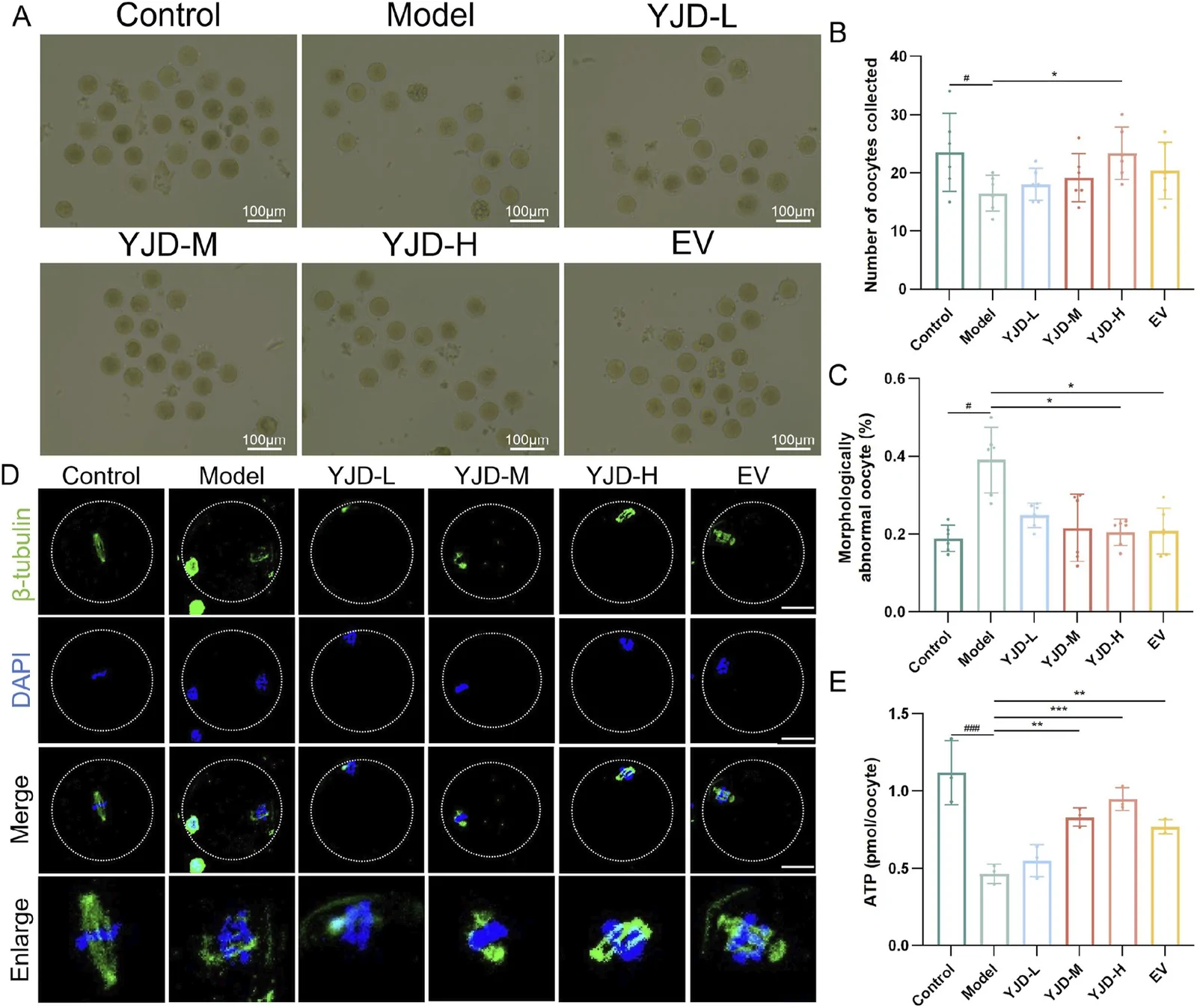
Effects of YJD on oocyte quality in DOR mice. **(A)** Representative images of MI oocytes in each group (scale bar: 100 μm). **(B)** Number of oocytes obtained via ovulation induction. **(C)** Percentage of abnormal oocytes. **(D)** Representative images of spindle staining in MI oocytes (scale bar: 20 μm). **(E)** ATP content. B, C: *n* = 6. E: *n* = 3. vs. Control: ^#^ *P* < 0.05, ^##^ *P* < 0.01, ^###^ *P* < 0.001; vs. Model: ^*^ *P* < 0.05, ^**^ *P* < 0.01, ^***^ *P* < 0.001.

### 3.4 YJD inhibits glycolysis and reduces lactate accumulation in MI oocytes of DOR mice

To investigate glycolysis in oocytes from mice with DOR, immunofluorescence was employed to assess the expression levels of key glycolytic proteins. The model group showed markedly increased expression levels of LDHA, GLUT1, and GLUT4 relative to the control group. Following treatment with medium-dose or high-dose YJD or estradiol valerate, LDHA expression was significantly reduced, and GLUT4 expression was markedly downregulated. GLUT1 expression was also significantly decreased following high-dose YJD and estradiol valerate intervention (Figures 4A–F). Under conditions of excessive glycolysis, intracellular glucose and pyruvate are ultimately converted to lactate by LDHA, resulting in lactate accumulation and consequent dysregulation of energy metabolism (Figure 4G). Consistent with this, our results demonstrated significantly increased pyruvate uptake and lactate production in MI oocytes isolated from DOR mice, indicating aberrant energy metabolism and enhanced glycolytic flux. Relative to the DOR model group, both the YJD-H and EV groups showed significantly reduced pyruvate uptake and decreased lactate production (Figures 4H,I). These findings suggest that high-dose YJD and EV may suppress glycolysis in MI oocytes and alleviate lactate accumulation.

**FIGURE 4:**
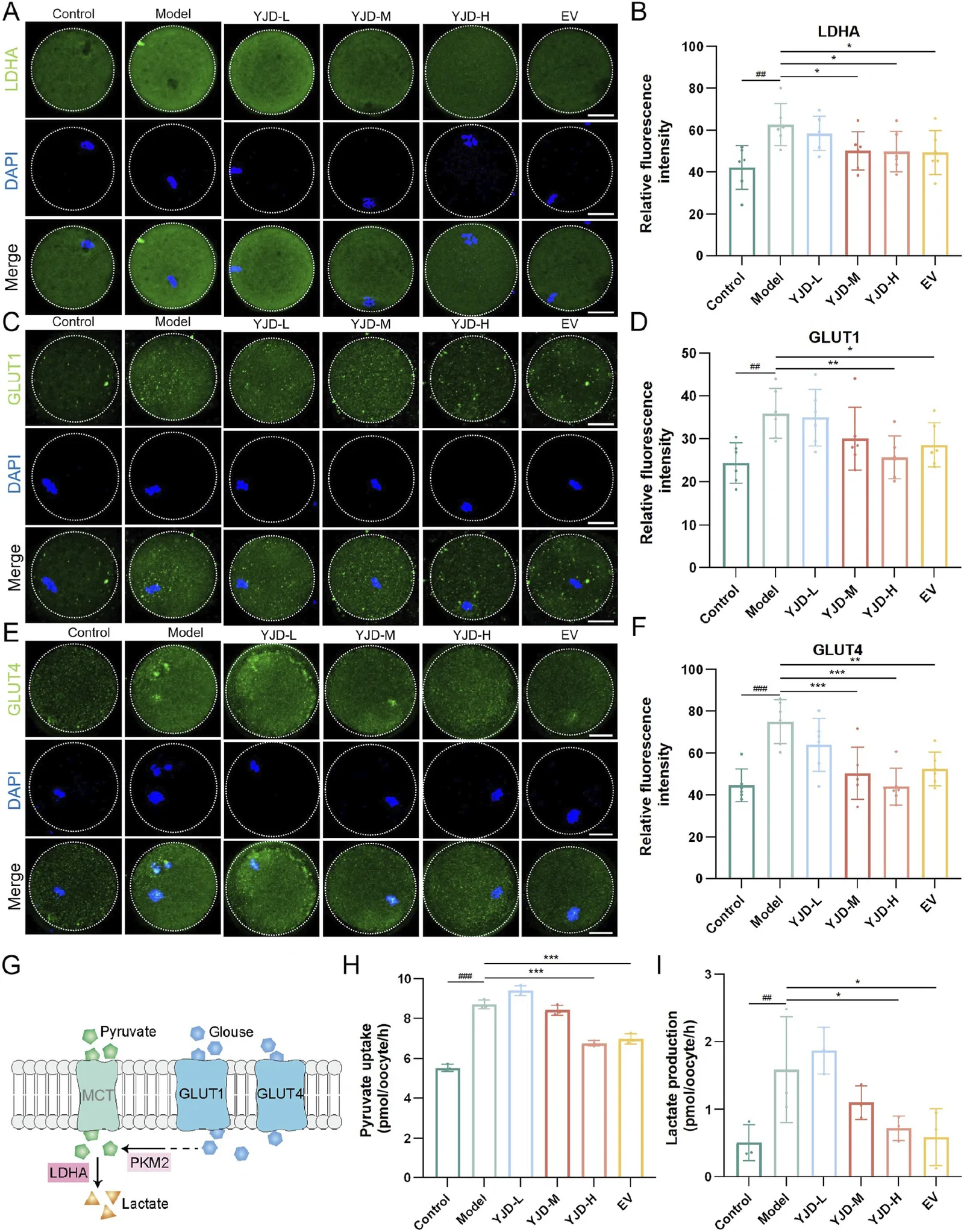
Effect of YJD on glycolytic protein expression and lactate accumulation in DOR mouse MI oocytes. **(A,C,E)** Representative images of LDHA, GLUT1, and GLUT4 fluorescence in MI oocytes of each group. (scale bar: 20 μm) **(B,D,F)** Relative fluorescence intensity of LDHA, GLUT1, and GLUT4. **(G)** Proteins associated with glycolysis mediate the uptake of pyruvate and the production of lactate. **(H)** Pyruvate intake. **(I)** Lactate production. B, D, F: *n* = 6 biologically independent oocytes, with one oocyte obtained from each of six different mice; H, I: *n* = 3 independent biological replicates, with 50 oocytes pooled per group in each replicate. vs. Control: ^#^ *P* < 0.05, ^##^ *P* < 0.01, ^###^ *P* < 0.001; vs. Model: ^*^ *P* < 0.05, ^**^ *P* < 0.01, ^***^ *P* < 0.001.

### 3.5 Regulation of SIRT3/HIF-1α/PDK1 protein expression in DOR oocytes by YJD

The SIRT3/HIF-1α/PDK1 axis constitutes a key regulatory pathway governing cellular lactate production (Lin, 2024; Ma et al., 2026). To assess its activity, we examined the protein expression levels of these components in MI oocytes from each experimental group via immunofluorescence. Results revealed that SIRT3 expression was significantly downregulated in MI oocytes isolated from DOR mice, whereas HIF-1α and PDK1 expression were both markedly upregulated. Notably, these aberrant expression patterns were effectively reversed following treatment with YJD-M, YJD-H, or EV (Figure 5). Collectively, these findings suggest that YJD improves oocyte energy metabolism, likely by modulating the SIRT3/HIF-1α/PDK1 pathway to suppress excessive lactate accumulation, thereby enhancing ATP generation and supporting proper spindle assembly and migration in MI oocytes from DOR mice.

**FIGURE 5:**
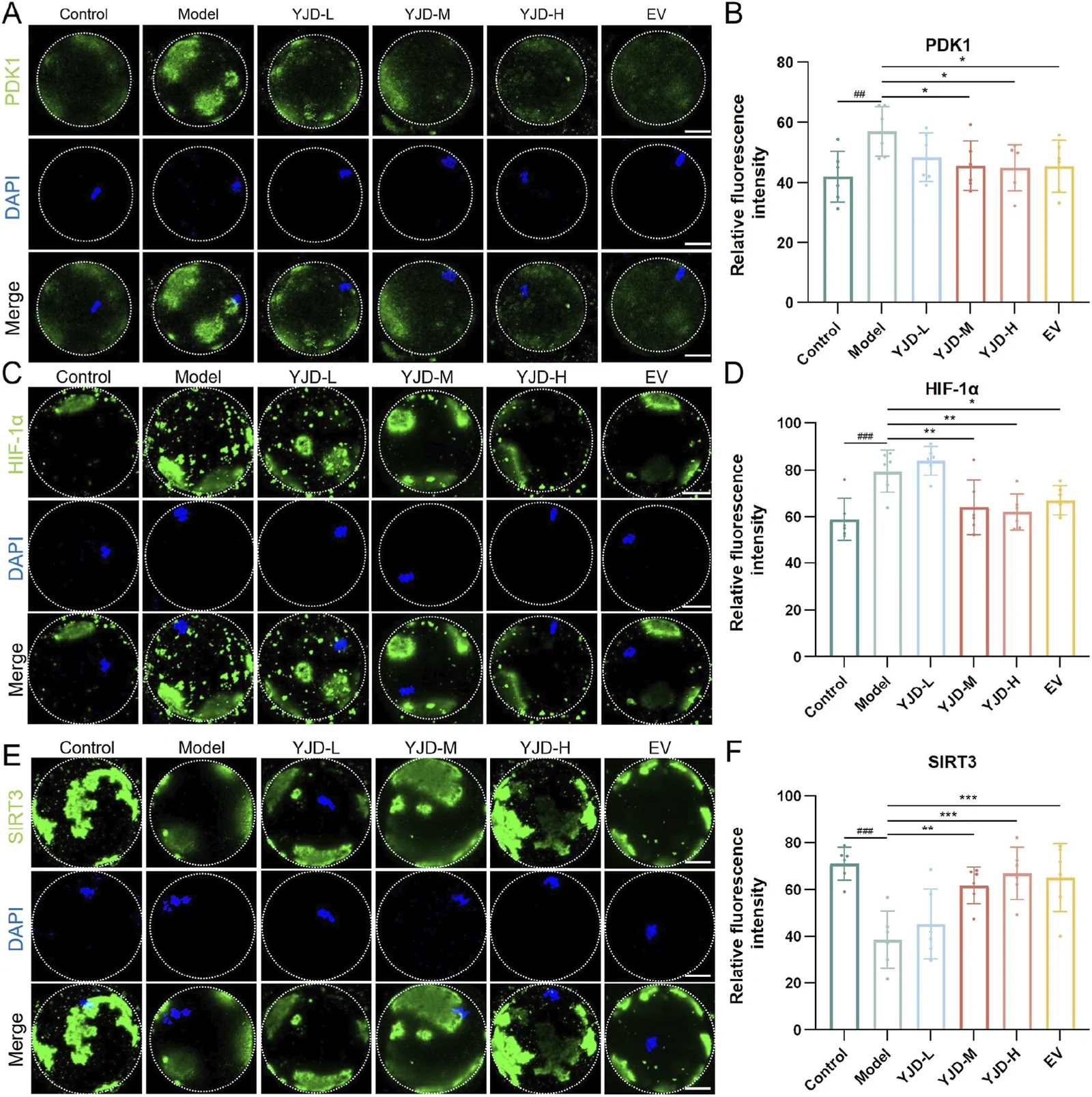
Impact of YJD on the SIRT3/HIF-1α/PDK1 signaling axis in MI oocytes from DOR mice. **(A,C,E)** Representative images of PDK1, HIF-1α, and SIRT3 fluorescence in MI oocytes of each group. (scale bar: 20 μm) **(B,D,F)** Relative fluorescence intensity of PDK1, HIF-1α, and SIRT3. B, D, F: *n* = 6 biologically independent oocytes, with one oocyte obtained from each of six different mice. vs. Control: ^#^ *P* < 0.05, ^##^ *P* < 0.01, ^###^ *P* < 0.001; vs. Model: ^*^ *P* < 0.05, ^**^ *P* < 0.01, ^***^ *P* < 0.001.

**FIGURE 6:**
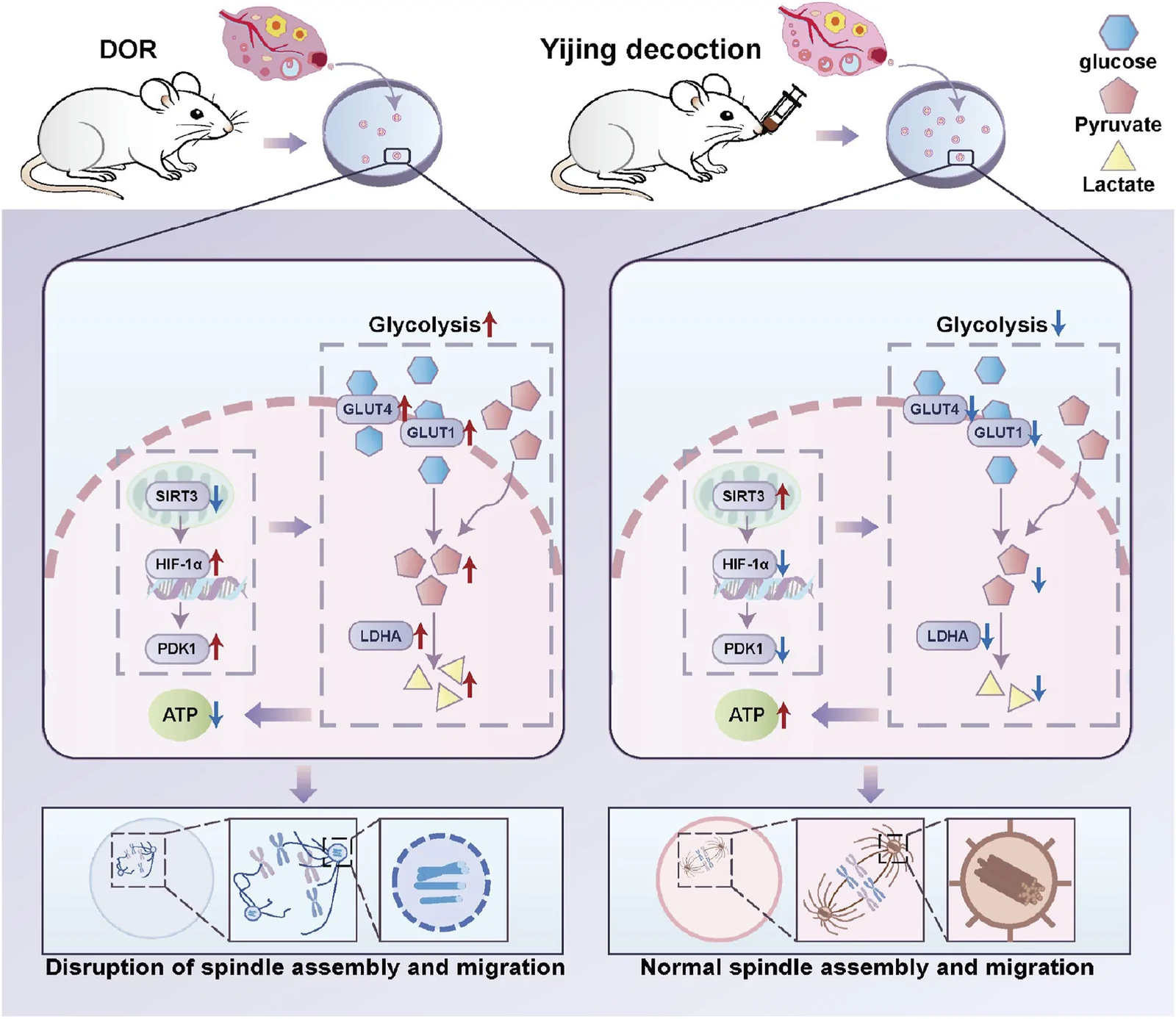
The mechanism diagram of YJD in treating DOR. YJD improves oocyte quality in DOR mice by alleviating lactate accumulation in oocytes through modulation of the SIRT3/HIF-1α/PDK1 signaling pathway.

## 4 Discussion

DOR is a degenerative reproductive disorder characterized by a decline in both the quantity and quality of oocytes, leading to a substantial impairment of female fertility. In recent years, the rising incidence of DOR has been attributed to multiple interrelated factors, including the societal trend toward delayed childbearing, increasing environmental pollution, adverse lifestyle modifications, and heightened psychological stress (Hao et al., 2023). Consequently, developing effective strategies to improve ovarian reserve has emerged as an urgent clinical priority. YJD is a classical traditional Chinese herbal formula that has long been used clinically for the management of ovarian insufficiency. Previous chemical characterization of YJD identified several blood-absorbed constituents with experimentally reported ovarian-protective activities. For example, paeoniflorin has been shown to improve ovarian reserve and granulosa-cell function and to promote mitophagy under oxidative stress (Wu et al., 2023; Xi et al., 2024), whereas ginsenoside Rg1 has been reported to preserve ovarian reserve by regulating mitochondrial homeostasis and PINK1/Parkin-mediated mitophagy (Yang et al., 2025). In addition, naringin and hesperidin have been shown to protect ovarian granulosa cells from cisplatin-induced injury (Liu et al., 2026; Abdeen et al., 2026). Despite the reported ovarian-protective activities of these blood-absorbed constituents and the long-standing clinical use of YJD, the molecular and physiological mechanisms underlying its beneficial effects in DOR remain to be fully elucidated.

The estrous cycle in mice reflects cyclical fluctuations in serum hormone levels and represents one of the simplest and reliable indicators for assessing ovarian function (Pouladvand et al., 2025). FSH, LH, and E_2_ collectively regulate follicular growth, development, and maturation, thereby serving as valuable biomarkers for evaluating ovarian reserve (Huang et al., 2022; ASRM, 2022). AMH exhibits a strong positive correlation with the number of small antral follicles and reliably reflects both the pool of early-stage growing follicles and the viability of primordial follicles. Consequently, AMH is widely regarded as an ideal marker for ovarian reserve assessment (Leiper et al., 2020; Wu et al., 2019). INHB, secreted predominantly by small antral follicles, modulates E_2_ production and provides complementary information on follicular health (Bedenk et al., 2020; Yding Andersen, 2017). Moreover, the number of follicles within the ovary constitutes a critical quantitative parameter for evaluating ovarian functional capacity (Huang, 2022). Importantly, oocyte spindle morphology, particularly during meiotic maturation, directly influences oocyte quality and subsequent embryonic developmental competence (Yue, 2024). Results from the present study demonstrate that YJD treatment effectively ameliorates multiple pathological features in DOR mice, including reduced ovarian index, disrupted estrous cyclicity, and aberrant serum hormone profiles. Furthermore, YJD promotes follicular development and maturation, suppresses follicular atresia, increases the number of MI oocytes, and improves oocyte spindle morphology. Results from the present study demonstrate that YJD treatment effectively ameliorates multiple pathological features in DOR mice, including reduced ovarian index, disrupted estrous cyclicity, and aberrant serum hormone profiles. Furthermore, YJD promotes follicular development and maturation, suppresses follicular atresia, increases the number of MI oocytes, and improves oocyte spindle morphology. Overall, most measured outcomes showed greater improvement with increasing YJD dose, suggesting a general dose-related therapeutic trend. However, this pattern was not uniform across all parameters, as the medium-dose group showed a greater improvement in serum LH levels than the high-dose group. This deviation may partly reflect the physiological pulsatility and variability of LH secretion, as well as the relatively limited sample size used for hormone measurements, which may increase the influence of inter-individual variation (Veldhuis et al., 1984). In addition, the dose–effect relationships of multi-component traditional Chinese medicine formulas may be complex and nonlinear (Li et al., 2025; Chen et al., 2022). Therefore, the present findings suggest an overall dose-related trend rather than a strictly linear or monotonic dose–response relationship. Collectively, these findings provide robust evidence that YJD exerts a significant protective effect on ovarian reserve function in DOR mice.

ATP synthesis is significantly enhanced during oocyte maturation, a process critical for accurate spindle assembly and chromosome segregation (Li et al., 2020). In oocytes, extracellular pyruvate and glucose enter the cell via specific transporters, including monocarboxylate transporters (MCTs), GLUT1, and GLUT4. Through glycolysis, these substrates generate modest amounts of ATP and are ultimately converted to lactate by LDHA (Zhang et al., 2024). However, due to its relatively low ATP yield per mole of substrate, glycolysis is transcriptionally and functionally suppressed during oocyte maturation; instead, mitochondrial oxidative phosphorylation becomes the dominant energy-producing pathway (Li, 2020). Recent evidence indicates that aberrant activation of glycolysis leads to pathological lactate accumulation, which disrupts folliculogenesis and compromises reproductive competence (Smits, 2023; Li et al., 2026). Excess lactate directly inhibits pyruvate dehydrogenase kinase two activity and stabilises the expression of key glycolytic enzymes, thereby reinforcing the conversion of pyruvate to lactate and ultimately diminishing mitochondrial ATP production. Concurrently, elevated lactate levels result in excessive generation of ROS. This ROS overload further damages mitochondrial integrity and function, exacerbating deficits in ATP synthesis (Rabinowitz and Enerbäck, 2020; Feng et al., 2022). Roberts et al. demonstrated an inverse correlation between meiotic progression rate and both pyruvate uptake and lactate production, such that faster meiotic resumption was associated with lower glycolytic flux and, consequently, less lactate accumulation (Roberts et al., 2002). Compared to in vivo–matured oocytes, IVM oocytes consistently exhibit elevated intracellular lactate concentrations, dysregulated energy metabolism, and markedly reduced developmental competence (Kirillova et al., 2021; Qin et al., 2022; Zhan et al., 2024). In contrast, culture media engineered to maintain low lactate levels effectively suppress ROS accumulation and enhance overall metabolic efficiency in oocytes (Soler et al., 2025). Consistent with these findings, the present study revealed significantly lower ATP levels in MI oocytes isolated from DOR mice relative to controls. Concomitant with this bioenergetic deficit, we observed abnormally elevated expression of the glycolytic proteins GLUT1, GLUT4, and LDHA, along with increased pyruvate uptake and lactate production, collectively indicating hyperactivation of glycolysis and excessive lactate accumulation. Notably, YJD intervention effectively downregulated the expression of these glycolysis-associated proteins, attenuated lactate accumulation, and restored ATP production.

PDK1 serves as a critical gatekeeper enzyme in glycolysis. By inhibiting pyruvate entry into the Tricarboxylic acid cycle, PDK1 enhances glycolytic flux (Li et al., 2024), resulting in cytoplasmic accumulation of pyruvate and its subsequent conversion to lactate (Park et al., 2024). In HaCaT-PIWIL2 cells, PDK1 knockdown markedly reduced the expression levels of key glycolytic enzymes, concomitant with significant decreases in glucose uptake and intracellular concentrations of glycolytic metabolites such as lactate (Li, 2024). HIF-1α is a master transcriptional regulator that drives the expression of multiple glycolytic genes. Under hypoxic conditions, HIF-1α protein expression is upregulated and translocates to the nucleus, where it dimerizes with HIF-1β to form the functional heterodimeric transcription factor HIF-1. This complex promotes the transcription of glycolytic effectors, including LDHA, glucose transporters GLUT1 and GLUT4, and PDK1 (Morris, 2022). Consistent with this, cellular studies demonstrate that HIF-1α overexpression activates glycolysis in NRK-49F cells and induces intracellular accumulation of both lactate and pyruvate (Hu et al., 2024). Multiple independent studies have reported abnormally elevated HIF-1α protein expression in the ovaries of DOR rodent models (Shu et al., 2026). SIRT3 modulates cellular metabolic reprogramming via the SIRT3–HIF-1α axis, particularly the shift from oxidative phosphorylation to glycolysis (Finley et al., 2011). Recent evidence indicates that SIRT3 inhibition leads to ROS accumulation, which in turn stabilizes and activates HIF-1α, thereby driving a metabolic switch toward glycolysis in neuronal cells (Ma, 2026). Clinically, SIRT3 mRNA levels are significantly lower in granulosa cells isolated from older pregnant women relative to younger women exhibiting normal ovarian reserve (Pacella-Ince et al., 2014). Similarly, SIRT3 protein expression is markedly reduced in oocytes from aged mice relative to age-matched controls (Yang et al., 2024). Collectively, these findings suggest that the SIRT3/HIF-1α/PDK1 signaling axis may constitute a pivotal regulatory pathway governing lactate homeostasis in DOR oocytes. The present study demonstrates that YJD treatment upregulates SIRT3 expression while concurrently suppressing HIF-1α and PDK1 expression in DOR mice. These results imply that YJD may alleviate excessive lactate accumulation in MI oocytes from DOR mice through modulation of the SIRT3/HIF-1α/PDK1 signaling pathway.

This study is subject to certain limitations. First, although preliminary data indicate that YJD modulates the SIRT3/HIF-1α/PDK1 signaling pathway and attenuates lactate accumulation in oocytes, rescue experiments, such as those employing SIRT3-knockout mice or specific pharmacological inhibitors of pathway components, were not performed. Consequently, direct causal evidence linking YJD’s effects to this pathway remains lacking. Second, due to the scarcity and technical challenges associated with isolating sufficient numbers of high-quality oocytes, we were unable to validate the expression levels of key pathway proteins and transcripts via RT-qPCR and Western blot analysis. Third, food consumption was not systematically quantified during the experiment; therefore, it remains unclear whether the observed changes in body weight were accompanied by alterations in food intake. Fourth, this study did not assess critical functional endpoints, including polar body extrusion at the MI stage or subsequent fertilization rates, thereby precluding a comprehensive evaluation of oocyte quality and developmental competence prior to fertilization. In addition, although the chemical profile and blood-penetrating constituents of YJD have been characterized and their putative herbal sources are provided in Supplementary Tables S1 and S2, the present study focused on evaluating YJD as an integral multi-component formula rather than identifying individual active compounds. Therefore, the specific bioactive constituents responsible for the observed effects and their direct relationships with therapeutic activity remain to be established. Future studies integrating rescue experiments, molecular and functional validation, longitudinal monitoring of food and water intake, and bioactivity-guided chemical investigation are warranted to further clarify the active chemical basis, mechanisms, and functional effects of YJD.

## 5 Conclusion

Overall, YJD treatment improved ovarian reserve function and MI oocyte quality in DOR mice. The alterations in lactate metabolism and the SIRT3/HIF-1α/PDK1 signaling pathway following YJD treatment may be associated with its protective effects on oocyte quality (Figure 6). These findings provide new insights into the potential association between lactate metabolism-related mechanisms and the protective effects of YJD against DOR and warrant further investigation.

## Data Availability

The original contributions presented in the study are included in the article/supplementary material, further inquiries can be directed to the corresponding authors.
