# Supplementary material for: Yijing Decoction improves oocyte quality and inhibits lactate accumulation in mice with diminished ovarian reserve
Source: Front Mol Biosci. 2026 Oct 15;13:1946851. doi: 10.3389/fmolb.2026.1946851 (PMC13716630; doi:10.3389/fmolb.2026.1946851)
Supplement: Supplementary file 1 [file Table1.docx]

Supplementary Material

# 1 Supplementary Figure 1

**Supplementary Figure 1.** **A** Representative estrous cycles of mice. P: Proestrus; E: Estrus; M: Metestrus; D: Diestrus. **B** The percent of regular and irregular estrous cycle. **C** Proportion of time spent in estrus. **D** The average days of estrous cycle. B, C, D: *n*=6. vs. Control: ^###^*P*< 0.001.

## 2 Supplementary Tables

**Supplementary Table 1.** The compounds were identified from YJD extract.

| NO. | Compounds | Rt(min) | Formula | Adducts | MS/MS | Observed mass(Da) | Calculated mass(Da) | PPM |
| --- | --- | --- | --- | --- | --- | --- | --- | --- |
| 1 | DL-Arginine | 0.74 | C_6_H_14_N_4_O_2_ | [M+H]^+^ | 175.1193, 158.0927, 116.0708, 70.0652 | 175.1193 | 175.119 | 1.71 |
| 2 | D-(-)-Mannitol | 0.8 | C_6_H_14_O_6_ | [M-H]^-^ | / | 181.0721 | 181.0718 | 1.66 |
| 3 | Rhamnose | 0.8 | C_6_H_14_O_6_ | [M-H]^-^ | 181.0721, 89.0245, 119.0352 | 181.0721 | 181.0718 | 1.66 |
| 4 | α, α-Trehalose | 0.83 | C_12_H_22_O_11_ | [M-H]^-^ | 341.1092, 179.0564, 161.0460, 119.0351, 101.0247, 89.0245, 71.0140 | 341.1092 | 341.1089 | 0.88 |
| 5 | D(+)-Glucose | 0.84 | C_6_H_12_O_6_ | [M-H]^-^ | 179.0565, 101.0244, 89.0245, 85.0297, 73.0295, 71.0140, 59.0139 | 179.0565 | 179.0561 | 2.23 |
| 6 | Sucrose | 0.88 | C_12_H_22_O_11_ | [M-H]^-^ | 341.1093, 179.0565, 161.0460, 119.0352, 101.0246, 89.0246, 71.0140, 59.0139 | 341.1093 | 341.1089 | 1.17 |
| 7 | Proline | 0.88 | C_5_H_9_NO_2_ | [M+H]^+^ | 116.0707, 70.0652 | 116.0707 | 116.0706 | 0.86 |
| 8 | L-(-)-Malic acid | 0.89 | C_4_H_6_O_5_ | [M-H]^-^ | 133.0145, 115.0039, 89.0247, 72.9933 | 133.0145 | 133.0142 | 2.26 |
| 9 | Adenine | 1.18 | C_5_H_5_N_5_ | [M+H]^+^ | 136.0620, 119.0358, | 136.062 | 136.0618 | 1.47 |
| 10 | Citric acid | 1.23 | C_6_H_8_O_7_ | [M-H]^-^ | 191.0201, 111.0090, 87.0089, 85.0297 | 191.0201 | 191.0197 | 2.09 |
| 11 | L-Tyrosine | 1.32 | C_9_H_11_NO_3_ | [M+H]^+^ | 182.0810, 165.0548, 136.0758, 123.0442, 91.0541 | 182.081 | 182.0812 | -1.1 |
| 12 | Isoleucine | 1.49 | C_6_H_13_NO_2_ | [M+H]^+^ | 132.1019, 86.0964, 69.0697 | 132.1019 | 132.1019 | 0 |
| 13 | Galloyl glucose | 1.81 | C_13_H_16_O_10_ | [M-H]^-^ | 331.0677, 271.0464, 169.0146 | 331.0677 | 331.0671 | 1.81 |
| 14 | Gallic acid | 1.92 | C_7_H_6_O_5_ | [M-H]^-^ | 169.0146, 125.0247, 107.0141, 97.0296, 69.0347 | 169.0146 | 169.0142 | 2.37 |
| 15 | L-Phenylalanine | 2.4 | C_9_H_11_NO_2_ | [M+H]^+^ | 166.0864, 131.0492, 120.0808, 103.0542 | 166.0864 | 166.0862 | 1.2 |
| 16 | Vanillic acid | 2.95 | C_8_H_8_O_4_ | [M-H]^-^ | 167.0352, 149.0243, 123.0455, 81.0347 | 167.0352 | 167.035 | 1.2 |
| 17 | Iso-geniposidic acid | 3 | C_16_H_22_O_10_ | [M-H]^-^ | 211.0615, 149.0610, 123.0454 | 373.1144 | 373.114 | 1.07 |
| 18 | Geniposidic acid | 3.26 | C_16_H_22_O_10_ | [M-H]^-^ | 211.0619, 149.0610, 123.0454, 167.0718 | 373.1144 | 373.114 | 1.07 |
| 19 | Mussaenosidic acid | 3.36 | C_16_H_24_O_10_ | [M-H]^-^ | 375.1304, 213.0771, 169.0874, 151.0766 | 375.1304 | 375.1297 | 1.87 |
| 20 | Protocatechuic acid | 3.46 | C_7_H_6_O_4_ | [M-H]^-^ | 153.0196, 109.0297, 108.0218, 91.0196 | 153.0196 | 153.0193 | 1.96 |
| 21 | Gentisic acid | 3.51 | C_7_H_6_O_4_ | [M-H]^-^ | 153.0196, 109.0297, 108.0220, 81.0347 | 153.0196 | 153.0193 | 1.96 |
| 22 | 6-Hydroxy geniposidic acid | 3.51 | C_16_H_22_O_11_ | [M-H]^-^ | 227.0565, 209.0463, 183.0666, 165.0560 | 389.1081 | 389.1089 | -2.06 |
| 23 | Deacetylasperulosidic acid | 3.51 | C_16_H_22_O_11_ | [M-H]^-^ | 345.1195, 227.0565, 183.0666, 165.0561, 139.0403 | 389.1081 | 389.1089 | -2.06 |
| 24 | Verbasoside | 3.57 | C_20_H_30_O_12_ | [M-H]^-^ | 461.1671, 297.0992, 315.1092, 161.0459, 153.0559, 135.0455, 89.0245, 73.0297, 59.0139 | 461.1671 | 461.1664 | 1.52 |
| 25 | 8-Epiloganic acid | 3.64 | C_16_H_24_O_10_ | [M-H]^-^ | 375.1301, 213.0776, 169.0873, 151.0768, 119.0353 | 375.1301 | 375.1297 | 1.07 |
| 26 | Neochlorogenic acid | 3.65 | C_16_H_18_O_9_ | [M-H]^-^ | 353.0883, 191.0565, 179.0354 | 353.0883 | 353.0878 | 1.42 |
| 27 | Aucubine | 3.88 | C_15_H_22_O_9_ | [M-H]^-^ | 345.1198, 179.0567, 89.0246, 59.0139 | 345.1198 | 345.1191 | 2.03 |
| 28 | 6-glc-coclaurine | 3.91 | C_23_H_29_NO_8_ | [M+H]^+^ | 237.0914, 175.0757, 137.0598, 209.0966 | 448.1971 | 448.1966 | 1.12 |
| 29 | Tryptophan | 3.93 | C_11_H_12_N_2_O_2_ | [M+H]^+^ | 205.0979, 159.0921, 144.0810, 118.0653 | 205.0979 | 205.0972 | 3.41 |
| 30 | Loganin | 4 | C_17_H_26_O_10_ | [M-H]^-^ | 89.0246, 389.1466, 59.0139, 101.0246, 85.0295 | 389.1466 | 389.1453 | 3.34 |
| 31 | Ortho-oxypeoniflorin | 4.25 | C_23_H_28_O_12_ | [M-H]^-^ | 495.1508, 137.0246, 93.0347 | 495.1508 | 495.1508 | 0 |
| 32 | Oxypeoniflorin | 4.53 | C_23_H_28_O_12_ | [M-H]^-^ | 495.1512, 165.0560, 137.0246, 93.0347 | 495.1512 | 495.1508 | 0.81 |
| 33 | Chlorogenic acid | 4.66 | C_16_H_18_O_9_ | [M-H]^-^ | 353.0882, 191.0564, 179.0358 | 353.0882 | 353.0878 | 1.13 |
| 34 | 7-Hydroxycoumarin | 4.78 | C_9_H_6_O_3_ | [M+H]^+^ | 163.0391, 145.0285, 117.0336, 107.0492, 89.0388 | 163.0391 | 163.039 | 0.61 |
| 35 | Chlorogenic acid | 4.79 | C_16_H_18_O_9_ | [M-H]^-^ | 353.0883, 191.0564, 179.0353 | 353.0883 | 353.0878 | 1.42 |
| 36 |  | 4.86 | C_11_H_12_O_6_ | [M-H]^-^ | 239.0566, 179.0354, 177.0562, 149.0612 | 239.0566 | 239.0561 | 2.09 |
| 37 | Syringic acid-4-O-α-L-rhamnoside | 5 | C_15_H_20_O_9_ | [M-H]^-^ | 343.1036, 299.1128, 284.0898, 197.0458 | 343.1036 | 343.1035 | 0.29 |
| 38 | Coclaurine | 5.14 | C_17_H_19_NO_3_ | [M+H]^+^ | 237.0911、209.0961、 191.0860、175.0755、 143.0492、107.0493 | 286.144 | 286.1438 | 0.7 |
| 39 | Paeonolide | 5.17 | C_20_H_28_O_12_ | [M+COOH]^-^ | 505.1557, 293.0884, 165.0560 | 505.1558 | 505.1551 | 1.39 |
| 40 | Caffeic acid | 5.26 | C_9_H_8_O_4_ | [M-H]^-^ | 179.0354, 135.0454, 107.0502 | 179.0354 | 179.035 | 2.23 |
| 41 | Homogentisic acid | 5.26 | C_8_H_8_O_4_ | [M-H]^-^ | 167.0353, 123.0454, 121.0299, 79.0554 | 167.0353 | 167.035 | 1.8 |
| 42 | Kaempferol-3-O-rutinoside | 5.31 | C_27_H_30_O_15_ | [M+H]^+^ | 595.1633、379.0819、337.0709、295.0603 | 595.1633 | 595.1657 | -4.03 |
| 43 | Purpureaside C | 5.48 | C_35_H_46_O_20_ | [M-H]^-^ | 785.2520, 161.0247, 623.2202 | 785.252 | 785.2509 | 1.4 |
| 44 | 1, 3, 6-Tri-O-galloyl-β-D-glucose | 5.5 | C_27_H_24_O_18_ | [M-H]^-^ | 635.0896, 465.0674, 165.0574 | 635.0896 | 635.089 | 0.94 |
| 45 | Echinacoside | 5.52 | C_35_H_46_O_20_ | [M-H]^-^ | 785.2520, 161.0247, 623.2199 | 785.252 | 785.2509 | 1.4 |
| 46 | N-Methylcoclaurine | 5.55 | C_20_H_24_NO_4_+ | [M]^+^ | 297.1125、282.0891、265.0862、237.0909、219.0805、191.0860 | 342.1702 | 342.1705 | -0.88 |
| 47 | Paeonilactone B | 5.65 | C_10_H_12_O_4_ | [M+H]^+^ | 197.0812, 179.0709, 151.0756 | 197.0812 | 197.0808 | 2.03 |
| 48 | Paeoniflorm | 5.97 | C_23_H_28_O_11_ | [M-H]^-^ | 479.1563, 449.1457, 327.1089, 165.0560, 121.0297 | 479.1563 | 479.1559 | 0.83 |
| 49 | Mudanpioside E | 6 | C_24_H_30_O_13_ | [M-H]^-^ | 525.1621, 479.1566, 449.1455, 327.1094, 165.0561, 121.0297 | 525.1621 | 525.1614 | 1.33 |
| 50 | JionosideA | 6.04 | C_36_H_48_O_20_ | [M-H]^-^ | 623.2202, 799.2679, 605.2086, 477.1611, 175.0404 | 799.2679 | 799.2666 | 1.63 |
| 51 | Neoaucubin | 6.07 | C_16_H_22_O_8_ | [M-H]^-^ | 179.0563, 143.0354, 119.0352 | 341.1251 | 341.1241 | 2.93 |
| 52 | Magnocurarine | 6.17 | C_19_H_24_NO_3_ | [M]^+^ | 237.0912、209.0959, 269.1176, 175.0757, 107.0493, 58.0653 | 314.1754 | 314.1756 | -0.64 |
| 53 | Vicenin-II | 6.23 | C_27_H_30_O_15_ | [M+H]^+^ | 595.1642、379.0818、337.0711、295.0604 | 595.1642 | 595.1657 | -2.52 |
| 54 | Isovitexin-2''-O-β-D-glucopyranoside | 6.27 | C_27_H_30_O_15_ | [M+H]^+^ | 595.1630、379.0815、337.0711、295.0599 | 595.1642 | 595.1657 | -2.52 |
| 55 | Vanillin | 6.4 | C_8_H_8_O_3_ | [M+H]^+^ | 153.0548, 111.0442, 93.0337 | 153.0548 | 153.0546 | 1.31 |
| 56 | 1, 2, 3, 6-Tetra-O-galloyl-β-D-glucose | 6.43 | C_34_H_28_O_22_ | [M-H]^-^ | 787.1007, 153.0193, 449.0732, 169.0145, | 787.1007 | 787.0999 | 1.02 |
| 57 | Spinosin | 6.48 | C_28_H_32_O_15_ | [M+H]^+^ | 447.1288、429.1180、351.0867、327.0866、297.0761、285.0767 | 609.1814 | 609.1814 | 0 |
| 58 | Safflomin A | 6.6 | C_27_H_32_O_16_ | [M-H]^-^ | 611.1624, 445.0992, 169.0145 | 611.1624 | 611.1618 | 0.98 |
| 59 | acteoside | 6.8 | C_29_H_36_O_15_ | [M-H]^-^ | 623.1988, 461.1666, 179.0354, 161.0247, 153.0560, 135.0453, 89.0245 | 623.1988 | 623.1981 | 1.12 |
| 60 | JionosideB | 6.82 | C_37_H_50_O_20_ | [M-H]^-^ | 637.2359, 813.2835, 619.2261, 491.1791, 175.0404 | 813.2835 | 813.2823 | 1.48 |
| 61 | 3-Hydroxycoumarin | 6.83 | C_9_H_6_O_3_ | [M+H]^+^ | 163.0390, 135.0442, 131.0492, 107.0493 | 163.0391 | 163.039 | 0.61 |
| 62 | paeonol | 6.91 | C_9_H_10_O_3_ | [M+H]^+^ | 167.0705, 149.0599, 121.0649 | 167.0705 | 167.0703 | 1.2 |
| 63 | Hymeromone | 6.94 | C_10_H_8_O_3_ | [M+H]^+^ | 134.0366, 89.0387, 149.0598, 177.0548 | 177.0548 | 177.0546 | 1.13 |
| 64 | Ferulic acid | 6.97 | C_10_H_10_O_4_ | [M-H]^-^ | 193.0511, 178.0275, 149.0610, 134.0376, 44.9983 | 193.0511 | 193.0509 | 1.04 |
| 65 | Galloylpaeoniflorin | 7 | C_30_H_32_O_15_ | [M-H]^-^ | 631.1675, 491.1196, 463.1245, 313.0572, 169.0146 | 631.1675 | 631.1668 | 1.11 |
| 66 | Fraxidin | 7.14 | C_11_H_10_O_5_ | [M+H]^+^ | 208.0370, 190.0263, 179.0348 | 223.0604 | 223.06 | 1.79 |
| 67 | Isoacteoside | 7.18 | C_29_H_36_O_15_ | [M-H]^-^ | 623.1989, 461.1669, 179.0354, 161.0247, 135.0454 | 623.1989 | 623.1981 | 1.28 |
| 68 | 4-O-galloylalbiflorin | 7.28 | C_30_H_32_O_15_ | [M-H]^-^ | 631.1676, 463.1249, 313.0573, 169.0146 | 631.1676 | 631.1668 | 1.27 |
| 69 | mudanpioside H | 7.39 | C_30_H_32_O_14_ | [M-H]^-^ | 615.1726, 281.0671, 137.0247 | 615.1726 | 615.1719 | 1.14 |
| 70 | Naringin | 7.45 | C_27_H_32_O_14_ | [M-H]^-^ | 579.1727, 459.1158, 271.0617, 151.0040 | 579.1727 | 579.1719 | 1.38 |
| 71 | Naringeninchalcone | 7.46 | C_15_ H_12_ O_5_ | [M+H]^+^ | 273.0760, 153.0184, 147.0442 | 273.076 | 273.0757 | 1.1 |
| 72 | Isochlorogenic acid A | 7.49 | C_25_H_24_O_12_ | [M-H]^-^ | 515.123 3, 353.0880, 191.0563, 179.0352, 135.0453 | 515.12 | 515.1194 | 1.16 |
| 73 | Narirutin | 7.51 | C_27_H_32_O_14_ | [M-H]^-^ | 579.1726, 459.1158, 271.0617, 151.0039 | 579.1726 | 579.1719 | 1.21 |
| 74 | 6'-O-galloylalbiflorin | 7.53 | C_30_H_32_O_15_ | [M-H]^-^ | 631.1674, 169.0146；313.0571 | 631.1674 | 631.1668 | 0.95 |
| 75 | 4, 5-Dicaffeoylquinic acid | 7.54 | C_25_H_24_O_12_ | [M-H]^-^ | 515.1204, 353.0883, 191.0565, 179.0353, 173.0457, 161.0247, 135.0454 | 515.1204 | 515.1195 | 1.75 |
| 76 | 6'''-p-Coumaroylspinosin | 7.57 | C_37_H_38_O_17_ | [M+H]^+^ | 429.1175、351.0865、327.0865、147.0441 | 755.2176 | 755.2181 | -0.66 |
| 77 | Rehmapicroside | 7.6 | C_16_H_26_O_8_ | [M-H]^-^ | 345.1557, 165.0924, 179.0564, 183.1035 | 345.1557 | 345.1555 | 0.58 |
| 78 | JionosideD | 7.6 | C_30_H_38_O_15_ | [M-H]^-^ | 637.2144, 461.1670, 315.1093, 193.0508, 135.0454 | 637.2144 | 637.2138 | 0.94 |
| 79 | Hesperetin | 7.86 | C_16_ H_14_ O_6_ | [M+H]^+^ | 303.0867, 177.0550, 153.0185 | 303.0867 | 303.0863 | 1.32 |
| 80 | Azelaic acid | 7.93 | C_9_H_16_O_4_ | [M-H]^-^ | 187.0979, 169.0874, 143.1080, 125.0974, 123.0818 | 187.0979 | 187.0976 | 1.6 |
| 81 | β-D-Glucopyranoside | 7.93 | C_23_H_28_O_11_ | [M-H]^-^ | 479.1564, 327.1083, 121.0297 | 479.1564 | 479.1559 | 1.04 |
| 82 | Eucommiol | 7.97 | C_9_H_16_O_4_ | [M-H]^-^ | 169.0872, 143.1081, 125.0974 | 187.0979 | 187.0975 | 2.14 |
| 83 | [Angulatin D](https://www.chembk.com/cn/chem/Angulatin D" \o "https://www.chembk.com/cn/chem/Angulatin D) | 8.11 | C_30_H_38_O_15_ | [M-H]^-^ | 637.2147, 461.1668, 315.1095, 193.0511, 135.0452 | 637.2147 | 637.2138 | 1.41 |
| 84 | 6-O-Feruloyl ajugol | 8.18 | C_25_H_32_O_12_ | [M-H]^-^ | 523.1798, 508.1591, 361.1276, 343.1163 | 523.1798 | 523.1821 | -4.4 |
| 85 | Gensenoside Rg1 | 8.25 | C_42_H_72_O_14_ | [M+COOH]^-^ | 845.4915, 637.4327, 475.3799, 131.0348, 101.0246, 89.0245 | 845.4915 | 845.4893 | 2.6 |
| 86 | Ginsenoside Re | 8.25 | C_48_H_82_O_18_ | [M+COOH]^-^ | 783.4894, 637.4327, 475.3801, 131.0357, 101.0248, 89.0248 | 991.5493 | 991.5472 | 2.12 |
| 87 | Salicylic acid | 8.31 | C_7_H_6_O_3_ | [M-H]^-^ | 137.0246, 93.0347 | 137.0246 | 137.0244 | 1.46 |
| 88 | Lactiflorin | 8.31 | C_23_H_26_O_10_ | [M+COOH]^-^ | 507.1510, 461.1456, 121.0297, 177.0561 | 507.151 | 507.1497 | 2.56 |
| 89 | Nornuciferine | 8.48 | C_18_H_19_NO_2_ | [M+H]^+^ | 282.1492、250.0992, 265.1226 | 282.1492 | 282.1488 | 1.42 |
| 90 | Isoliquiritin | 8.58 | C_31_H_40_O_15_ | [M-H]^-^ | 651.2302, 175.0404, 475.1819, 193.0509 | 651.2302 | 651.2294 | 1.23 |
| 91 | mudanpioside C | 8.92 | C_30_H_32_O_13_ | [M-H]^-^ | 137.0246, 281.0661, 599.1775, 121.0296 | 599.1775 | 599.177 | 0.83 |
| 92 | Rehmannioside | 9 | C_31_H_40_O_15_ | [M-H]^-^ | 651.2301, 175.0403, 475.1818, 193.0509 | 651.2301 | 651.2294 | 1.07 |
| 93 | Isofraxidin | 9.16 | C_11_H_10_O_5_ | [M+H]^+^ | 223.0604, 208.0376, 179.0333 | 223.0604 | 223.06 | 1.79 |
| 94 | Moutanpioside J | 9.17 | C_31_H_34_O_14_ | [M-H]^-^ | 629.1882, 121.0298, 137.0246, | 629.1882 | 629.1876 | 0.95 |
| 95 | 8, 9-Epoxyatractylenolide | 10.06 | C_20_H_36_O_7_ | [M+H]^+^ | 389.2548, 371.2445, 311.2121 | 389.2548 | 389.2533 | 3.85 |
| 96 | benzoylpaeoniflorin | 10.17 | C_30_H_32_O_12_ | [M+COOH]^-^ | 629.1886, 553.1724, 121.0297 | 629.1886 | 629.1865 | 3.34 |
| 97 | Ginsenoside Rf | 10.24 | C_42_H_72_O_14_ | [M+COOH]^-^ | 799.4861, 475.3802, 131.0360, 101.0247, 89.0246 | 845.4914 | 845.4893 | 2.48 |
| 98 | Quinquenoside L11 | 10.24 | C_42_H_72_O_14_ | [M+COOH]^-^ | 845.4914, 637.4334, 475.3802, 131.0360, 101.0247, 89.0246 | 845.4914 | 845.4893 | 2.48 |
| 99 | Benzoylalbiflorin | 10.34 | C_30_H_32_O_12_ | [M+COOH]^-^ | 629.1885, 553.1721, 121.0297 | 629.1885 | 629.1865 | 3.18 |
| 100 | Ginsenoside Rb1 | 10.51 | C_54_H_92_O_23_ | [M-H]^-^ | / | 1107.5978 | 1107.5957 | 1.9 |
| 101 | Ginsenoside F3 | 10.57 | C_41_H_70_O_13_ | [M+COOH]^-^ | 815.4813, 637.4327, 475.3797, 131.0351, 101.0246, 89.0245 | 815.4813 | 815.4787 | 3.19 |
| 102 | Majonoside R1 | 10.57 | C_42_H_72_O_15_ | [M-H]^-^ | 815.4813, 191.0566, 131.0352, 101.0246, 89.0245 | 815.4813 | 815.4798 | 1.84 |
| 103 | Notoginsenoside A | 10.77 | C_54_H_92_O_24_ | [M-H]^-^ | 1123.5919, 131.0356, 101.0248, 89.0248 | 1123.5919 | 1123.5905 | 1.25 |
| 104 | Notoginsenoside R2 | 10.83 | C_41_H_70_O_13_ | [M+COOH]^-^ | 815.4813, 191.0577, 131.0353, 101.0246, 89.0246 | 815.4813 | 815.4787 | 3.19 |
| 105 | Ginsenoside F5 | 10.85 | C_41_H_70_O_13_ | [M+COOH]^-^ | 815.4814, 637.4330, 475.3795, 131.0353, 101.0246, 89.0246 | 815.4814 | 815.4787 | 3.31 |
| 106 | Ginsenoside Ro | 10.93 | C_48_H_76_O_19_ | [M-H]^-^ | 955.4918, 793.4390, 731.4388, 659.4184, 613.3748, 569.3856, 455.353 | 955.4918 | 955.4908 | 1.05 |
| 107 | Chikusetsu saponin IV | 10.99 | C_48_H_76_O_19_ | [M-H]^-^ | 955.4917, 731.4382, 659.4139, 613.3748, 569.3856, 455.3528 | 955.4917 | 955.4908 | 0.94 |
| 108 | Atractylenolide VI | 11.02 | C_15_H_22_ | [M+H]^+^ | 203.1798, 161.1329, 147.1171 | 203.1798 | 203.1794 | 1.97 |
| 109 | Saikosaponin C | 11.03 | C_48_H_78_O_17_ | [M+COOH]^-^ | 971.5236, 925.5176, 779.4596, 763.4647, 617.4052 | 971.5236 | 971.521 | 2.68 |
| 110 | Ginsenoside Rh1 | 11.05 | C_36_H_62_O_9_ | [M+COOH]^-^ | 683.4386, 637.4295, 475.3803, 101.0249 | 683.4386 | 683.4365 | 3.07 |
| 111 | Ginsenoside F1 | 11.25 | C_36_H_62_O_9_ | [M+COOH]^-^ | 683.4387, 637.4307, 475.3800, 101.0244, 89.0247 | 683.4387 | 683.4365 | 3.22 |
| 112 | Ginsenoside Rd | 11.61 | C_48_H_82_O_18_ | [M+COOH]^-^ | 783.4922, 459.3858, 131.0357 | 991.5493 | 991.5472 | 2.12 |
| 113 | Acetovanillone | 11.64 | C_9_H_10_O_3_ | [M+H]^+^ | 167.0705, 149.0601, 121.0654 | 167.0705 | 167.0703 | 1.2 |
| 114 | Gypenoside XVII | 11.68 | C_48_H_82_O_18_ | [M+COOH]^-^ | 621.4381, 459.3845, 783.4917 | 991.5491 | 991.5472 | 1.92 |
| 115 | Saikosaponin A | 12.51 | C_42_H_68_O_13_ | [M+COOH]^-^ | 825.4648, 779.4595, 617.4062 | 825.4648 | 825.4631 | 2.06 |
| 116 | Saikosaponin D | 13.16 | C_42_H_68_O_13_ | [M+COOH]^-^ | 825.4654, 779.4599, 617.4064 | 825.4654 | 825.4631 | 2.79 |
| 117 | Dilactam A | 13.63 | C_15_H_21_NO | [M+H]^+^ | 232.1699, 145.1017, 95.0857 | 232.1699 | 232.1696 | 1.29 |
| 118 | Dinbutylphthalate | 13.64 | C_16_H_22_O_4_ | [M+H]^+^ | 279.1596, 149.0236, 191.1070, 233.1541 | 279.1596 | 279.1591 | 1.79 |
| 119 | Zingibroside R1 | 13.84 | C_42_H_66_O_14_ | [M+COOH]^-^ | 793.4390, 631.3882, 455.3531, 157.0151, 113.0247 | 793.439 | 793.438 | 1.26 |
| 120 | Chikusetsu saponin IVa | 13.9 | C_42_H_66_O_14_ | [M-H]^-^ | 793.4390, 455.3534, 157.0145, 113.0247 | 793.439 | 793.438 | 1.26 |
| 121 | Atractylenolide I | 14.2 | C_15_H_18_O_2_ | [M+H]^+^ | 231.1383, 203.1437, 189.0915, 163.0756, 119.0856, 105.0699 | 231.1383 | 231.138 | 1.3 |
| 122 | Ginsenoside F4 | 14.41 | C_42_H_70_O_12_ | [M+H]^+^ | 767.4953, 133.1016, 85.0286 | 767.4953 | 767.494 | 1.69 |
| 123 | Betulin | 14.42 | C_30_H_50_O_2_ | [M+H]^+^ | 443.3886, 425.3780, 407.3689 | 443.3886 | 443.3884 | 0.45 |
| 124 | Ginsenoside Rg5 | 14.57 | C_42_H_70_O_12_ | [M+H]^+^ | 767.4951, 133.1014, 85.0286 | 767.4951 | 767.494 | 1.43 |
| 125 | Z-ligustilide | 15.28 | C_12_H_14_O_2_ | [M+H]^+^ | 191.1070, 173.0964, 145.1015, 91.0543, 79.0545, 67.0543 | 191.107 | 191.1067 | 1.57 |
| 126 | Ginsenoside Rs3 | 15.75 | C_44_H_74_O_14_ | [M+COOH]^-^ | 621.4365, 459.3853, 101.0246 | 871.5069 | 871.505 | 2.18 |
| 127 | Ligustilide | 15.83 | C_12_H_14_O_2_ | [M+H]^+^ | 191.1069, 173.0964, 145.1015, 107.0488, 91.0543, 79.0543, 67.0542 | 191.1069 | 191.1067 | 1.05 |
| 128 | Atractylenolide II | 16.48 | C_15_H_20_O_2_ | [M+H]^+^ | 233.1540, 215.1434, 187.1485, 133.1015 | 233.154 | 233.1536 | 1.72 |
| 129 | 3β-Hydroxyatractylone | 16.48 | C_15_H_20_O_2_ | [M+H]^+^ | 205.1591, 197.1332, 187.1485, 159.0806, 151.0755, 105.0701, 95.0855 | 233.154 | 233.1536 | 1.72 |
| 130 | Ginsenoside Rg6 | 16.61 | C_42_H_70_O_12_ | [M+H]^+^ | 767.4927, 605.4443, 85.0284 | 767.4927 | 767.494 | -1.69 |
| 131 | arachidic acid | 16.65 | C_20_H_40_O_2_ | [M+NH4]^+^ | 253.2337, 183.0822, 181.1439, 155.0751, 153.0971 | 330.3372 | 330.3366 | 1.82 |
| 132 | GinsenosideCK | 17.54 | C_36_H_62_O_8_ | [M+COOH]^-^ | / | 667.4443 | 667.4416 | 4.05 |
| 133 | GinsenosideRh3 | 17.77 | C_36_H_62_O_8_ | [M+COOH]^-^ | / | 667.4434 | 667.4416 | 2.7 |
| 134 | Conferone | 19.08 | C_24_H_28_O_4_ | [M+H]^+^ | 381.2065, 191.1069, 173.0964, 163.1116, 145.1015, 107.0494, 91.0544 | 381.2065 | 381.206 | 1.31 |
| 135 | Majonoside R2 | 19.31 | C_41_H_70_O_14_ | [M-H]^-^ | 787.4874, 475.3813, 131.0355, 101.0248, 89.0248 | 787.4874 | 787.4838 | 4.57 |
| 136 | 3-butylphthalide | 19.32 | C_12_H_14_O_2_ | [M+H]^+^ | 191.1069, 173.0963, 145.1016, 91.0543, 79.0544, 67.0541 | 191.1069 | 191.1067 | 1.05 |
| 137 | Levistolide A | 19.41 | C_24_H_28_O_4_ | [M+H]^+^ | 381.2065, 191.1069, 173.0965, 163.1120, 145.1014, 135.0446, 107.0492, 91.0544 | 381.2065 | 381.206 | 1.31 |
| 138 | 4-phenoxycyclohexanone | 19.54 | C_12_H_14_O_2_ | [M+H]^+^ | 191.1069, 173.0965, 145.1016, 91.0544, 79.0544, 67.0542 | 191.1069 | 191.1067 | 1.05 |
| 139 | Angelicide | 19.63 | C_24_H_28_O_4_ | [M+H]^+^ | 381.2066, 191.1069, 173.0967, 163.1121, 145.1012, 135.0444, 107.0494, 91.0544 | 381.2066 | 381.206 | 1.57 |
| 140 | senkyunolide O | 20.8 | C_24_H_28_O_4_ | [M+H]^+^ | 381.2066, 191.1069, 173.0964, 163.1121, 145.1014, 107.0494, 91.0544 | 381.2066 | 381.206 | 1.57 |
| 141 | Oleanolic acid | 21.69 | C_30_ H_48_O_3_ | [M-H]^-^ | / | 455.3539 | 455.3535 | 0.88 |
| 142 | Ursolic acid | 21.71 | C_30_ H_48_O_3_ | [M-H]^-^ | / | 455.3539 | 455.3535 | 0.88 |
| 143 | Erucamide | 24.06 | C_22_H_43_NO | [M+H]^+^ | 338.3421, 321.3155, 303.3055 | 338.3421 | 338.3417 | 1.18 |
| 144 | Stearic Acid | 25.72 | C_18_H_36_O_2_ | [M-H]^-^ | 283.2647 | 283.2647 | 283.2643 | 1.41 |
| 145 | Ginsenoside Rk1 | 27.69 | C_42_H_70_O_12_ | [M+COOH]^-^ | 765.4804, 101.0246, 89.0246 | 811.4866 | 811.4838 | 3.45 |

**Supplementary Table 2.** The compounds identified from YJD serum.

| NO. | Compounds | Rt(min) | Formula | Adducts | MS/MS | Observed mass(Da) | Calculated mass(Da) | PPM |
| --- | --- | --- | --- | --- | --- | --- | --- | --- |
| 1 | 8-Epiloganic acid | 3.61 | C_16_H_24_O_10_ | [M-H]^-^ | / | 375.1303 | 375.1297 | 1.6 |
| 2 | Aucubin | 3.82 | C_15_H_22_O_9_ | [M-H]^-^ | / | 345.12 | 345.1191 | 2.61 |
| 3 | Loganin | 4.01 | C_17_H_26_O_10_ | [M-H]^-^ | / | 389.1464 | 389.1453 | 2.83 |
| 4 | Oxypaeoniflorin | 4.52 | C_23_H_28_O_12_ | [M-H]^-^ | / | 495.1517 | 495.1508 | 1.82 |
| 5 | Coclaurine | 5.16 | C_17_H_19_NO_3_ | [M+H]^+^ | / | 286.1439 | 286.1438 | 0.35 |
| 6 | N-Methylcoclaurine | 5.59 | C_20_H_24_NO_4_^+^ | [M]^+^ | / | 342.1701 | 342.1705 | -1.17 |
| 7 | Paeoniflorin | 5.98 | C_23_H_28_O_11_ | [M-H]^-^ | / | 479.1566 | 479.1559 | 1.46 |
| 8 | Mudanpioside E | 5.98 | C_24_H_30_O_13_ | [M-H]^-^ | 525.1615，479.1562，449.1455，327.1087，165.0559，121.0297 | 525.1615 | 525.1614 | 0.19 |
| 9 | Neoaucubin | 6.02 | C_16_H_22_O_8_ | [M-H]^-^ | / | 341.1246 | 341.1241 | 1.47 |
| 10 | Mudanpioside H | 7.39 | C_30_H_32_O_14_ | [M-H]^-^ | / | 615.1733 | 615.1719 | 2.28 |
| 11 | Naringin | 7.45 | C_27_H_32_O_14_ | [M-H]^-^ | / | 579.1725 | 579.1719 | 1.04 |
| 12 | Narirutin | 7.5 | C_27_H_32_O_14_ | [M-H]^-^ | / | 579.1726 | 579.1719 | 1.21 |
| 13 | Rehmapicroside | 7.66 | C_16_H_26_O_8_ | [M-H]^-^ | / | 345.1558 | 345.1555 | 0.87 |
| 14 | Eucommiol | 7.95 | C_9_H_16_O_4_ | [M-H]^-^ | / | 187.0979 | 187.0975 | 2.14 |
| 15 | Ginsenoside Rg1 | 8.27 | C_42_H_72_O_14_ | [M+COOH]^-^ | / | 845.4926 | 845.4893 | 3.9 |
| 16 | Lactiflorin | 8.31 | C_23_H_26_O_10_ | [M+COOH]^-^ | / | 507.1512 | 507.1497 | 2.96 |
| 17 | Mudanpioside C | 8.97 | C_30_H_32_O_13_ | [M-H]^-^ | / | 599.1765 | 599.177 | -0.83 |
| 18 | Benzoylpaeoniflorin | 10.17 | C_30_H_32_O_12_ | [M+COOH]^-^ | / | 629.1884 | 629.1865 | 3.02 |
| 19 | Benzoylalbiflorin | 10.36 | C_30_H_32_O_12_ | [M+COOH]^-^ | / | 629.188 | 629.1865 | 2.38 |
| 20 | Ginsenoside Ro | 10.93 | C_48_H_76_O_19_ | [M-H]^-^ | / | 955.4906 | 955.4908 | -0.21 |
| 21 | Ginsenoside Rh1 | 11.01 | C_36_H_62_O_9_ | [M+COOH]^-^ | / | 683.4396 | 683.4365 | 4.54 |
| 22 | Chikusetsu saponin IV | 11.04 | C_48_H_76_O_19_ | [M-H]^-^ | / | 955.4906 | 955.4908 | -0.21 |
| 23 | Ginsenoside F1 | 11.24 | C_36_H_62_O_9_ | [M+COOH]^-^ | / | 683.4371 | 683.4365 | 0.88 |
| 24 | Ginsenoside Rd | 11.59 | C_48_H_82_O_18_ | [M+COOH]^-^ | / | 991.5476 | 991.5472 | 0.4 |
| 25 | Saikosaponin A | 12.53 | C_42_H_68_O_13_ | [M+COOH]^-^ | / | 825.4659 | 825.4631 | 3.39 |
| 26 | Saikosaponin D | 13.13 | C_42_H_68_O_13_ | [M+COOH]^-^ | / | 825.4656 | 825.4631 | 3.03 |
| 27 | Atractylenolide I | 14.2 | C_15_H_18_O_2_ | [M+H]^+^ | / | 231.1381 | 231.138 | 0.43 |
| 28 | Betulin | 14.42 | C_30_H_50_O_2_ | [M+H]^+^ | / | 443.3871 | 443.3884 | -2.93 |
| 29 | Ginsenoside F4 | 14.42 | C_42_H_70_O_12_ | [M+H]^+^ | / | 767.4919 | 767.494 | -2.74 |
| 30 | Ginsenoside Rg6 | 16.69 | C_42_H_70_O_12_ | [M+H]^+^ | / | 767.4956 | 767.494 | 2.08 |
| 31 | Oleanolic acid | 21.68 | C_30_ H_48_ O_3_ | [M-H]^-^ | / | 455.3535 | 455.3535 | 0 |
| 32 | Ursolic acid | 22.74 | C_30_ H_48_ O_3_ | [M-H]^-^ | / | 455.3534 | 455.3535 | -0.22 |
